# Relationship between the turbidity difference and the grade of green tea under Ca^2+^ acceleration: A preliminary study

**DOI:** 10.1002/fsn3.2974

**Published:** 2022-08-09

**Authors:** Xiao‐Lan Yu, Yong He

**Affiliations:** ^1^ College of Biosystems Engineering and Food Science Zhejiang University Hangzhou China

**Keywords:** Ca^2+^ acceleration, green tea grade, turbidity difference

## Abstract

The grade of green tea indicates its intrinsic quality and guides consumers when purchasing. Simple, accessible, and on‐site determination of green tea grades is essential for consumers and regulators. In this study, we assumed that the turbidity difference in green tea might indicate its grade, and our results confirmed this hypothesis. The turbidity difference was measured in green tea infusions before and after the Ca^2+^ acceleration. For the same kind of green tea, it was found that higher grades of green tea had larger turbidity differences. Effects of brewing temperature, brewing time, Ca^2+^ concentration, and Ca^2+^ treatment time on the turbidity of green tea infusions were analyzed, and their optimal values were obtained. This study demonstrates that applying the turbidity difference and Ca^2+^ acceleration could be an accessible method for the on‐site determination of green tea grades.

## Graphical abstract

For the same kind of green tea, the higher grade, the larger the turbidity difference of green tea infusions before and after the Ca^2+^ acceleration. Effects of green tea brewing temperature, green tea brewing time, Ca^2+^ concentration, and Ca^2+^ treatment time were analyzed and optimal values were selected. This study demonstrates that applying the turbidity difference and Ca^2+^ acceleration could be an accessible method for the on‐site determination of green tea grades.

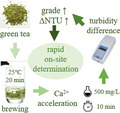

## 1 INTRODUCTION

On‐site, accessible, and simple determination of green tea grades is the development trend of tea quality and safety. Sensory evaluation of tea is the standard method to determine the grade of tea. This method mainly relies on the appearance of tea leaves, the tenderness of raw tea leaves, and the color, the aroma, and the taste of tea infusions (GB/T 23776‐2018, 2018; Han et al., 2022). Sensory evaluation of tea is performed by qualified professionals, and tea samples are evaluated in a standard evaluation room using standard evaluation utensils (Yu et al., 2020); thus, this method is not accessible for the on‐site determination of tea grades. Furthermore, for the same kind of green tea, huge price differences exist between its different grades. For instance, the price (per 500 g) of the highest grade of Xihu Longjing—one of the top 10 popular teas in China—is 15–20 times the price of its lowest grade (Yu et al., 2009; Yu, Yao et al., 2008; Yu, Zhang et al., 2008).

Although tea grade is evaluated by the sensory evaluation method, it is essentially determined by the chemical compositions of tea leaves and the combined visual, olfactory, and taste sensations induced by these chemical compositions. Tea cream, which is the visible haze and precipitate that forms spontaneously in a hot tea infusion on cooling (Dickinson, 1994), is the early process of precipitation in the tea infusion system (Xu & Yin, 2010). It was first recognized in black tea and is actually common in various categories of tea, including fermented and unfermented teas (Kim & Talcott, 2012; Lin et al., 2015). The chemical components in a tea infusion affect the amount of tea cream remarkably. It has been shown that the main components of tea cream in green tea infusions are polyphenols (29.86%–78.66%), total sugar (14.47%–27.62%), and caffeine (2.35%–10.43%) (Yin et al., 2009). In polyphenols, catechins accounted for the most, and in catechins, (‐)‐epigallocatechin (EGC), (‐)‐epigallocatechin gallate (EGCG), (‐)‐epicatechin (EC), and (‐)‐epicatechin gallate (ECG) participated mostly. Recent studies (Chen et al., 2019; Couzinet‐Mossion et al., 2010; Ishizu et al., 2016) are focused on understanding the formation mechanism of tea cream and determining a method to reduce its formation to ensure the clarity of black and green tea beverages and extend their shelf life.

The components that form green tea cream are closely related to those that affect the taste of green tea infusions, and taste is one of the most important factors in determining the quality of green tea. Moreover, for consumers, the taste is the foremost drinking experience of green tea. Therefore, we proposed a hypothesis that the turbidity difference, which indicates the amount of tea cream formed in a certain period of time, might indicate the grade of green tea. Turbidity is the measure of the relative clarity of a liquid. Tea infusions and beverages become turbid when tea cream appears, and then their turbidity increases with time. Researchers have applied turbidity to measure the clarity of tea beverages or tea infusions (Bindes et al., 2019; Chen et al., 2019; Guo et al., 2021; Kim & Talcott, 2012). Considering the acceleration effect of Ca^2+^ on the formation of green tea cream (Chen et al., 2019; Xu et al., 2013) and the purpose of rapid determination of green tea grades, Ca^2+^ acceleration was used to verify the hypothesis mentioned above.

In this study, we chose turbidity and analyzed the effects of green tea brewing temperature, green tea brewing time, Ca^2+^ concentration, and Ca^2+^ treatment time on the turbidity of green tea infusions, respectively. The purpose of this study was to investigate the feasibility of using the turbidity difference as an indicator of the grade of green tea and establish a simple and accessible method for determining the green tea with the higher grade in the field.

## 2 MATERIALS AND METHODS

### 2.1 Materials

Different grades of four types of green teas, Xihu Longjing, Qiantang Longjing, Huangshan Maofeng, and Anji Baicha, were bought and used. All of them were products of geographical indications in China, and their information is presented in detail in Table 1. Xihu Longjing labeled A and B, as well as Qiantang Longjing labeled D and E were picked and produced by different tea companies in the same year, and Xihu Longjing labeled B and C, as well as Qiantang Longjing labeled E and G were picked and produced by the same tea company in different years. It should be noted that the grades in Table 1 are self‐determined by the tea companies, and are only valid for green tea produced and sold by the same tea company, and the grades between different tea companies are not comparable. All the green teas were picked before the solar term Qingming, which is around April 5th of every year. Calcium chloride, dihydrate, in the analytical grade were bought from Sinopharm Chemical Reagents Co., Ltd. For the convenience of the experiment, the commercially available Watsons distilled water was used in the experiments.

**TABLE 1:** Information of green teas in this study

Commercial name	Grade	Label	Price (RMB/100 g)
Xihu Longjing	AAA	A1	432
	AA	A2	280
	A	A3	186
Xihu Longjing	First‐class	B1	272
	Second‐class	B2	196
Xihu Longjing	First‐class	C1	272
	Second‐class	C2	196
Qiantang Longjing	First‐class	D1	240
	Second‐class	D2	80
Qiantang Longjing	Second‐class	E1	65
	Third‐class	E2	36
Qiantang Longjing	Special‐class	F1	480
	First‐class	F2	270
Qiantang Longjing	First‐class	G1	72
	Second‐class	G2	39.6
Huangshan Maofeng	First‐class	H1	280
	Second‐class	H2	130
Anji Baicha	5‐star	I1	456
	4‐star	I2	316
	3‐star	I3	196

### 2.2 Analysis of green tea brewing temperature

Both room temperature (25°C) and boiling water were analyzed to investigate the effect of brewing temperature on the turbidity of green tea infusions with respect to time. The standing time of green tea infusions was 1, 24, 48, 72, 96, and 120 h. The ratio of green tea and brewing water was 1:50 (g:ml). At room temperature, green tea leaves were filtered after 1 h, while at the boiling water, green tea leaves were brewed for 5 min and then filtered. Tea infusions were placed open during the standing process.

### 2.3 Analysis of green tea brewing time

The green tea brewing time was 5, 10, 20, 30, 45, and 60 min to evaluate the effect of brewing time on the turbidity of green tea infusions. The brewing temperature was obtained from the result of *Analysis of green tea brewing temperature* in Section 2.2.

### 2.4 Analysis of Ca^2+^ concentration

In the green tea infusion after brewing, the Ca^2+^ concentration was 0, 100, 200, 300, 400, 500, and 600 mg/L. The brewing temperature and brewing time were acquired from the results of the analyses mentioned above, and the treatment time of Ca^2+^ was set as 10 min.

### 2.5 Analysis of Ca^2+^ treatment time

To understand the effect of Ca^2+^ treatment time on the turbidity of green tea infusions, different treatment times of 0, 5, 10, 15, 20, and 30 min were set. Green tea brewing temperature, green tea brewing time, and Ca^2+^ concentration were acquired from the results of the analyses mentioned above, respectively.

### 2.6 Turbidity

The turbidity of each green tea infusion was measured using a portable turbidity meter WGZ‐500B (Shanghai Xinrui Instruments Co., Ltd.).

The green tea sample E1 (Huangshan Maofeng with the first‐class) was used for analyzing the effects of green tea brewing temperature, green tea brewing time, Ca^2+^ concentration, and Ca^2+^ treatment time on the turbidity of green tea infusions. The turbidity differences were calculated from the measured turbidities of the green tea infusions.

### 2.7 Determination of chemical components in green tea

The contents of catechins, EGC, EC, EGCG, and ECG, in green tea samples were determined using the national standard method (GB/T 8313‐2018, 2018). Moreover, the contents of free amino acids (GB/T 8314‐2013, 2013) and caffeine (GB/T 8312‐2013, 2013) were also determined using the national standard methods.

The catechins, EGC, EC, ECGC, and ECG, in the ground tea samples were extracted with a 70% methanol aqueous solution in a water bath at 70°C. Their determination used a C18 column (250 mm × 4.6 mm, 5 μm), a detection wavelength of 278 nm, gradient elution, and high‐pressure liquid chromatography (HPLC) analysis. The mixtures of ethylenediamine tetraacetic acid (EDTA)‐2Na:acetic acid:acetonitrile:water (1:10:45:444, v/v/v) and EDTA‐2Na:acetic acid:acetonitrile:water (1:10:400:89, v/v/v) were used as mobile phases A and B, respectively. The gradient program was as follows: 100% solvent A for 10 min, solvent A from 100% to 68% by the linear gradient in 15 min, 68% solvent A for 10 min, and then 100% solvent A. External standard method was used for the direct quantification.

The caffeine in the ground tea samples was extracted with the hot water in the boiling water bath. Its determination used a C18 column (octadecyl silane), a detection wavelength of 280 nm, and HPLC analysis. The mixture of methanol:water (3:7, v/v) was used as the mobile phase. External standard method was used for the direct quantification.

The ninhydrin method was used to determine the content of α‐amino acids. Amino acids were coheated with ninhydrin under at pH 8.0 to form a purple complex. The absorbance of the obtained complex was measured at 570 nm, and the content was calculated from the standard curve.

### 2.8 Data analysis

The contents of catechins, free amino acids, and caffeine of green tea samples, and the turbidity of green tea infusions were measured three times for each sample. The mean and standard deviation were calculated from the three values of each parameters. One‐way analysis of variance (ANOVA) test was conducted to assess statistically significant difference with 0.05 as the significant level.

## 3 RESULTS AND DISCUSSION

### 3.1 Effects of green tea brewing temperature

**FIGURE 1:**
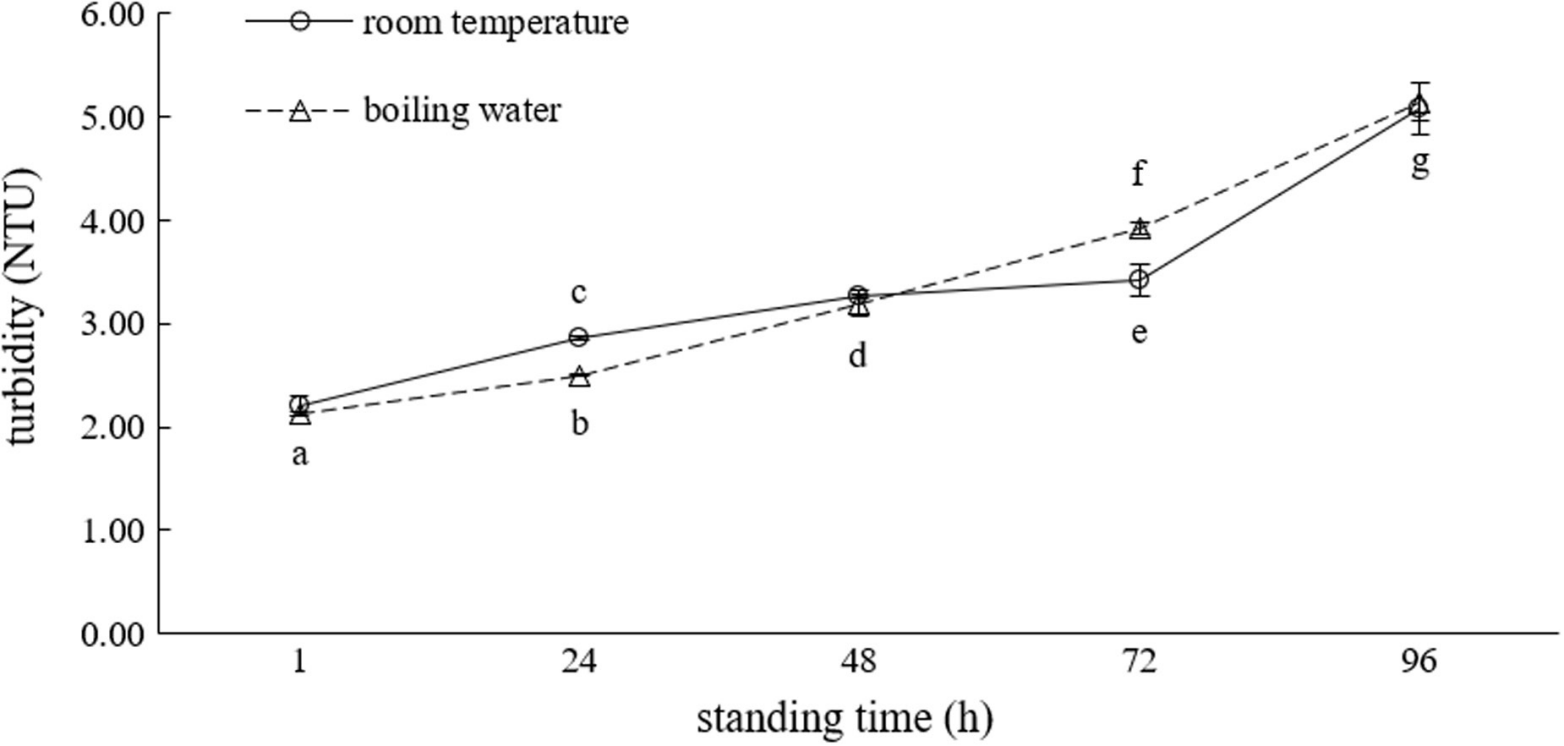
Effects of green tea brewing temperature on the turbidity of green tea infusions

With the trend of cold brew (Liu et al., 2017; Song et al., 2021) on the rise, consumers generally use boiling water to brew green tea. The brewing temperature affects the concentration of the components, such as caffeine (Liu et al., 2017), in the tea infusion, as well as the aroma and taste of the tea infusion. When a green tea infusion is stood for certain days, the brewing temperature has no effect on its turbidity, as shown in Figure 1.

It was found that the turbidity of both green tea infusions increased with increasing standing time; however, their increasing trends were not consistent. Significant differences occurred at the standing time of 24 h and 72 h; however, when the standing time was added to 96 h, no significant difference between the two brewing temperatures was observed, which was same for the standing time of 1 h. At the standing time of 120 h, molds appeared in the green tea infusions; thus, the turbidity of both green tea infusions was not measured.

Considering the experimental results and the convenience of experiments, the room temperature, which was 25°C in this study, was used as the brewing temperature for the following experiments.

### 3.2 Effects of green tea brewing time

**FIGURE 2:**
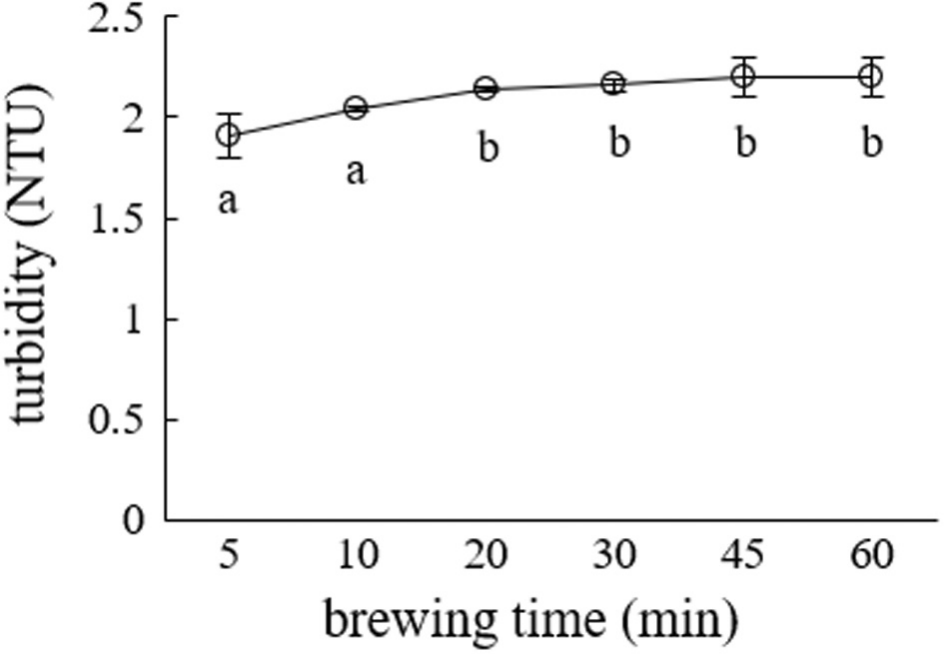
Effects of green tea brewing time on the turbidity of green tea infusions

Brewing time affected the turbidity of green tea infusions at the brewing temperature of 25°C, as seen in Figure 2. In the initial brewing time of 5–20 min, the turbidity of the green tea infusion increased with increasing brewing time. When the brew time increased to 60 min, no significant change in the turbidity was observed. This observation suggests that when green tea was brewed at 25°C, its extracts reached the maximum of turbidity in 20 min and stabilized thereafter. Therefore, for green tea, 20 min was selected as the brewing time at the brewing temperature of 25°C.

### 3.3 Effects of Ca^2+^ concentration and Ca^2+^ treatment time

**FIGURE 3:**
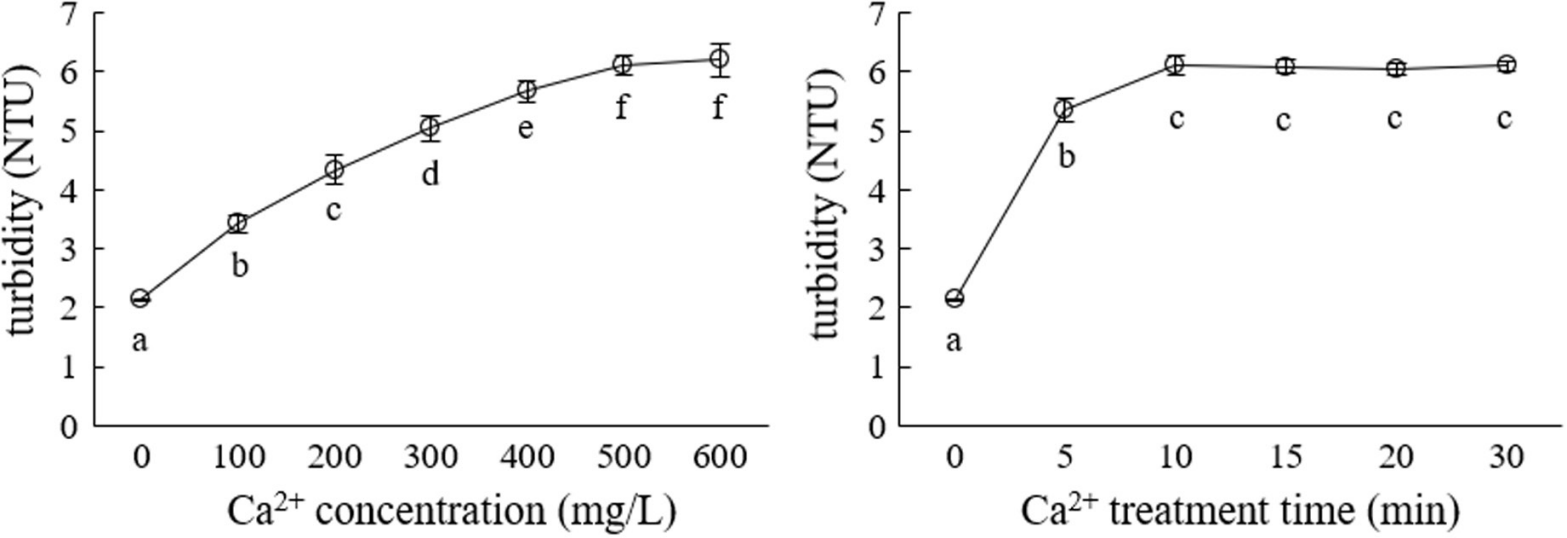
Effects of Ca^2+^ concentration and Ca^2+^ treatment time on the turbidity of green tea infusions

Figure 3 shows the effects of Ca^2+^ concentration and Ca^2+^ treatment time on the turbidity of green tea infusions.

Consistent with existing researches (Chen et al., 2019; Xu et al., 2013), Ca^2+^ had an accelerating effect on the formation of green tea cream, which was reflected by the addition of the turbidity of green tea infusions. As shown in Figure 3, the effects of Ca^2+^ concentration and Ca^2+^ treatment time on the turbidity of green tea infusions were similar, that is, the turbidity increased at first and then stabilized. In the range of 0–500 mg/L, with the increase in Ca^2+^ concentration, the turbidity of the green tea infusion increased. When the Ca^2+^ concentration further increased to 600 mg/L, no significant change in the turbidity of the green tea infusion was observed. For 0–10 min, the increase in the Ca^2+^ treatment time increased the turbidity of the green tea infusion; however, with the further increase in the treatment time after 10 min, no significant difference in the turbidity was observed. Therefore, the optimal values of Ca^2+^ concentration and Ca^2+^ treatment time were selected as 500 mg/L and 10 min, respectively.

### 3.4 Correlation between turbidity differences and green tea grades

**FIGURE 4:**
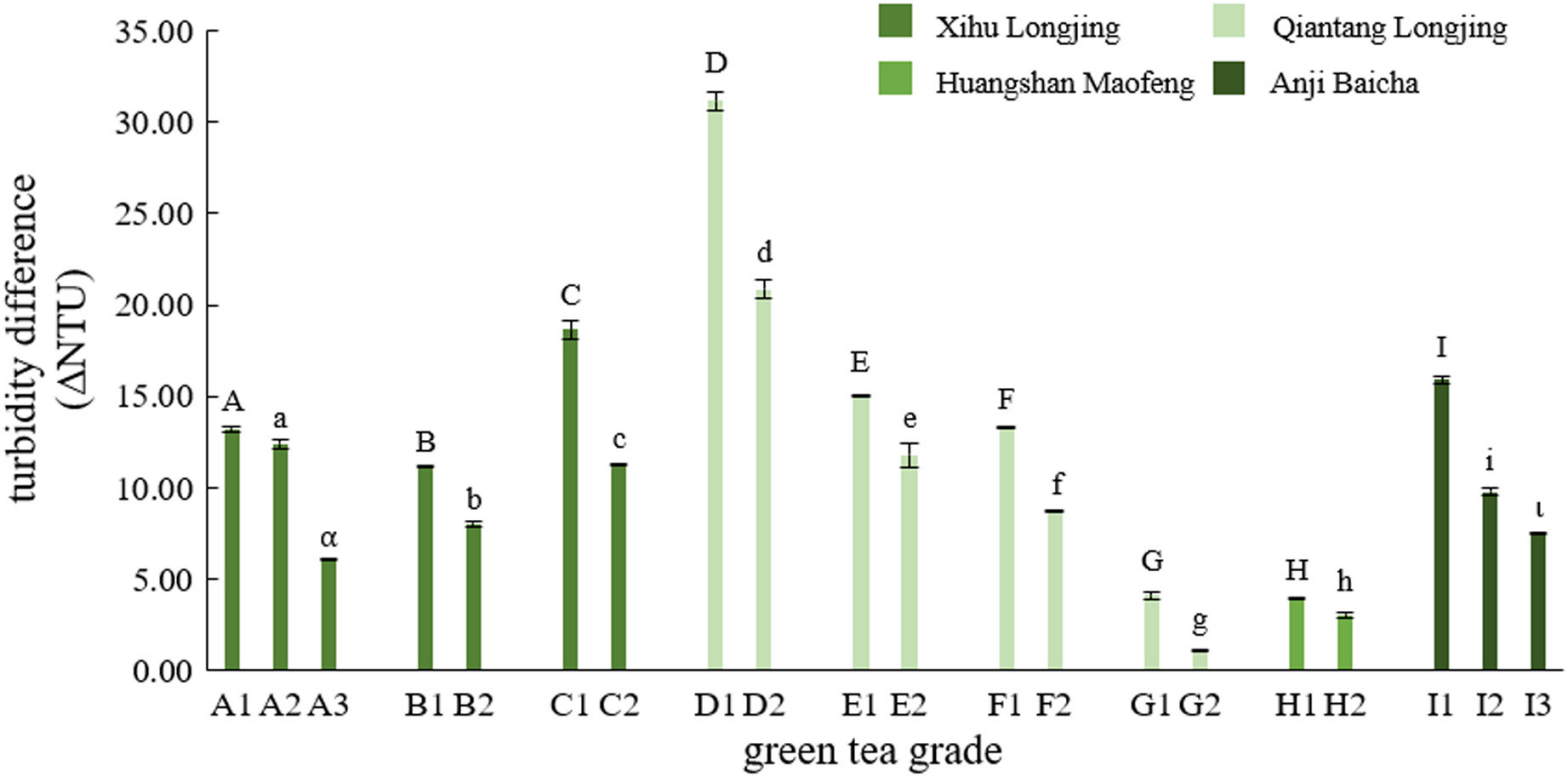
Turbidity differences of green teas with different grades

**FIGURE 5:**
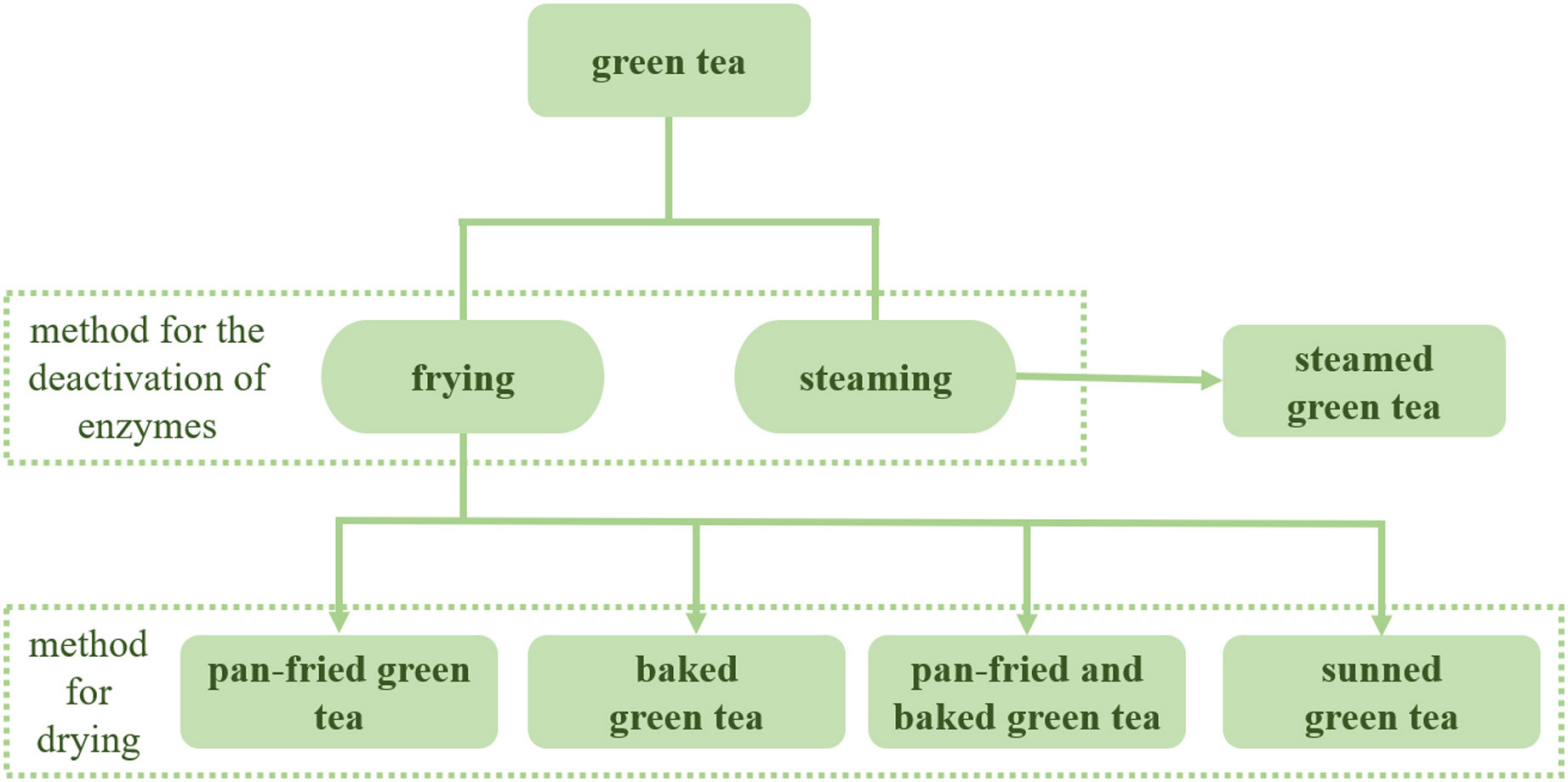
Classification of green tea

**FIGURE 6:**
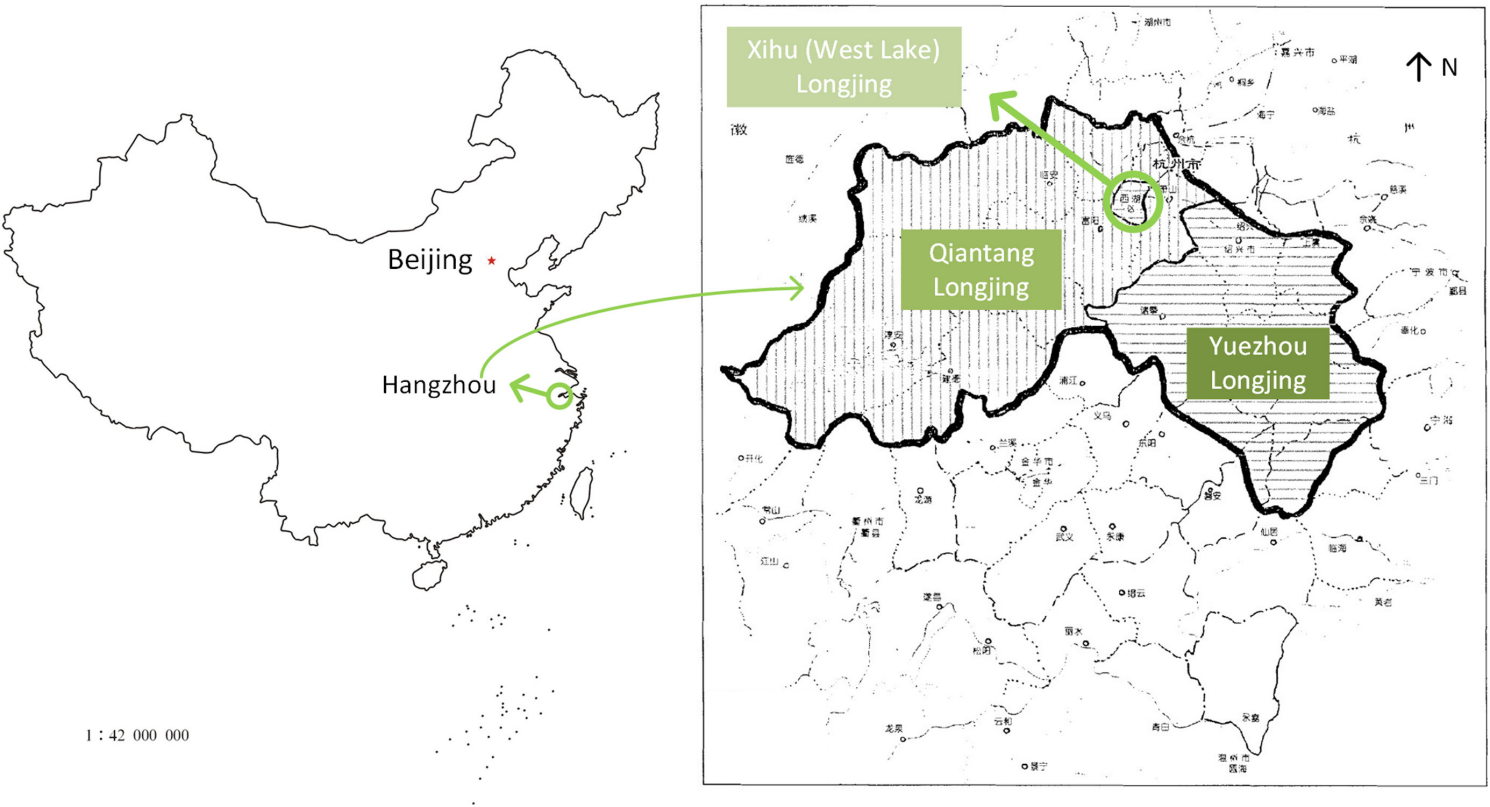
The geographical origins of Longjing tea

Under the experimental conditions (i.e., brewing temperature = 25°C, brewing time = 20 min, Ca^2+^ concentration = 500 mg/L, and Ca^2+^ treatment time = 10 min), no obvious correlation between the turbidities before and after the Ca^2+^ acceleration of green tea samples was observed. However, it was found that the difference in turbidities before and after Ca^2+^ acceleration correlated with the grade of a green tea, that is, higher grade had a larger turbidity difference, as shown in Figure 4. The uppercase and lowercase of the same letter in Figure 4 indicate a significant difference. This is true for all types of green tea used in this study, that is, Xihu Longjing, Qiantang Longjing, Huangshan Maofeng, and Anji Baicha. Among the four types of green teas used in this study, Xihu Longjing and Qiantang Longjing are pan‐fried green tea, Huangshan Maofeng is baked green tea, and Anji Baicha is pan‐fried and baked green tea, whose differences lie in the method for drying, seen in Figure 5. As for Xihu Longjing and Qiantang Longjing, their differences exist in the area where the tea trees were grown (GB/T 18650‐2008, 2008), as Figure 6 illustrates.

It is noteworthy that, except the grade, other parameters of the green tea being compared (with the same capital letter, for example, A1, A2, and A3 in Figure 4) were the same, such as the processing factory, the area where the tea trees were grown, the variety of tea trees, and the year of tea leaves picked and made. But once other parameters are the same, this study discovered that the higher the grade of the same kind of green tea, the larger the turbidity difference before and after Ca^2+^ acceleration. The relationship between the grade and the difference in turbidity before and after Ca^2+^ acceleration of the same kind of green tea is not affected by the difference in the processing factory (A and B in Figure 4, as well as D and E) or the year in which the tea was picked and made (B and C in Figure 4, as well as E and G). This relationship demonstrates the feasibility of using turbidity difference to reflect green tea grades. Applying the turbidity difference and Ca^2+^ acceleration treatment made the on‐site determination of green tea grades accessible, compared to the sensory evaluation of tea. Larger sample size in terms of types and grades (for each type) of green teas is required to test the universality of the correlation between the turbidity difference and grades of a green tea. A database of turbidity differences of green teas with known grades is worth establishing, and it will be conducive to the rapid and on‐site determination of green tea grades.

### 3.5 Chemical components related to the formation of green tea cream and green tea grade

**FIGURE 7:**
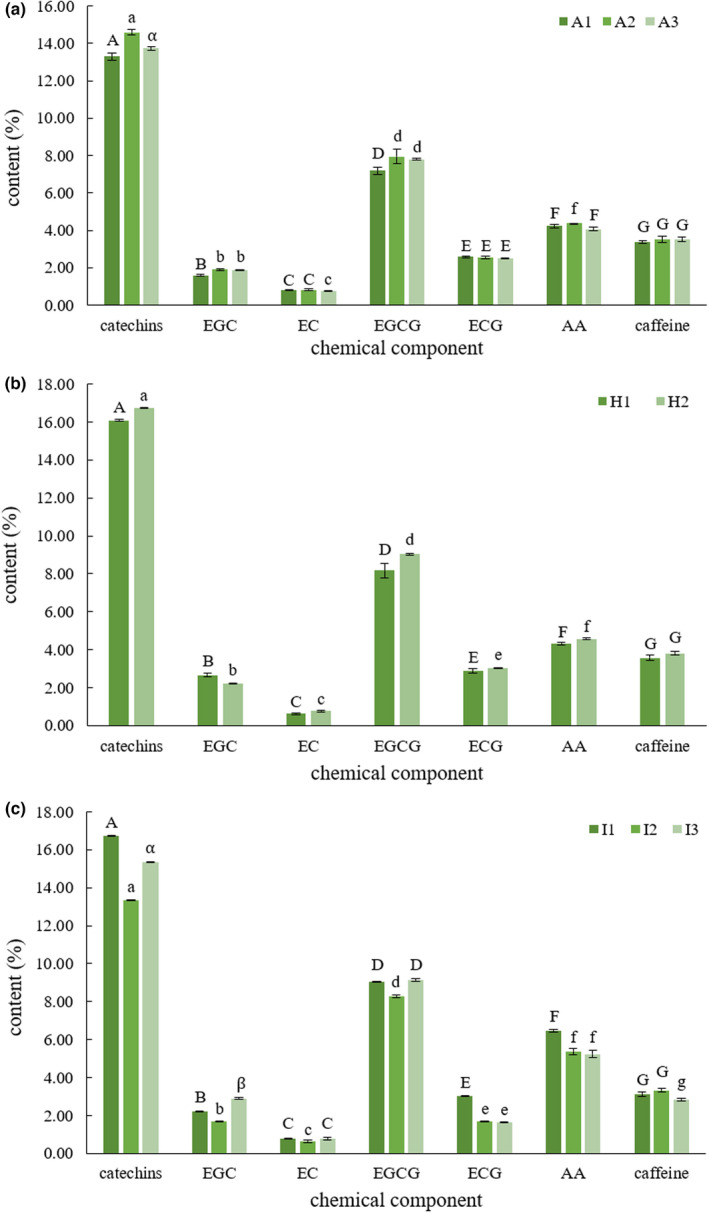
Chemical components contents of Xihu Longjing, Huangshan Maofeng, and Anji Baicha

The different grades of green tea undergo the same processing technology (Han et al., 2022), though, significant differences exist between the contents of chemical components, as seen in Figure 7, however, there is no consistency in the variation relationship of chemical components' contents between different grades of the same kind of green tea. In this figure, green tea samples A1, A2, and A3 (Xihu Longjing) represent pan‐fried green tea, H1 and H2 (Huangshan Maofeng) represent baked green tea, and I1, I2, and I3 (Anji Baicha) represent pan‐fried and baked green tea. The uppercase and lowercase of the same letter indicate that significant differences exist between the contents of chemical components.

**FIGURE 8:**
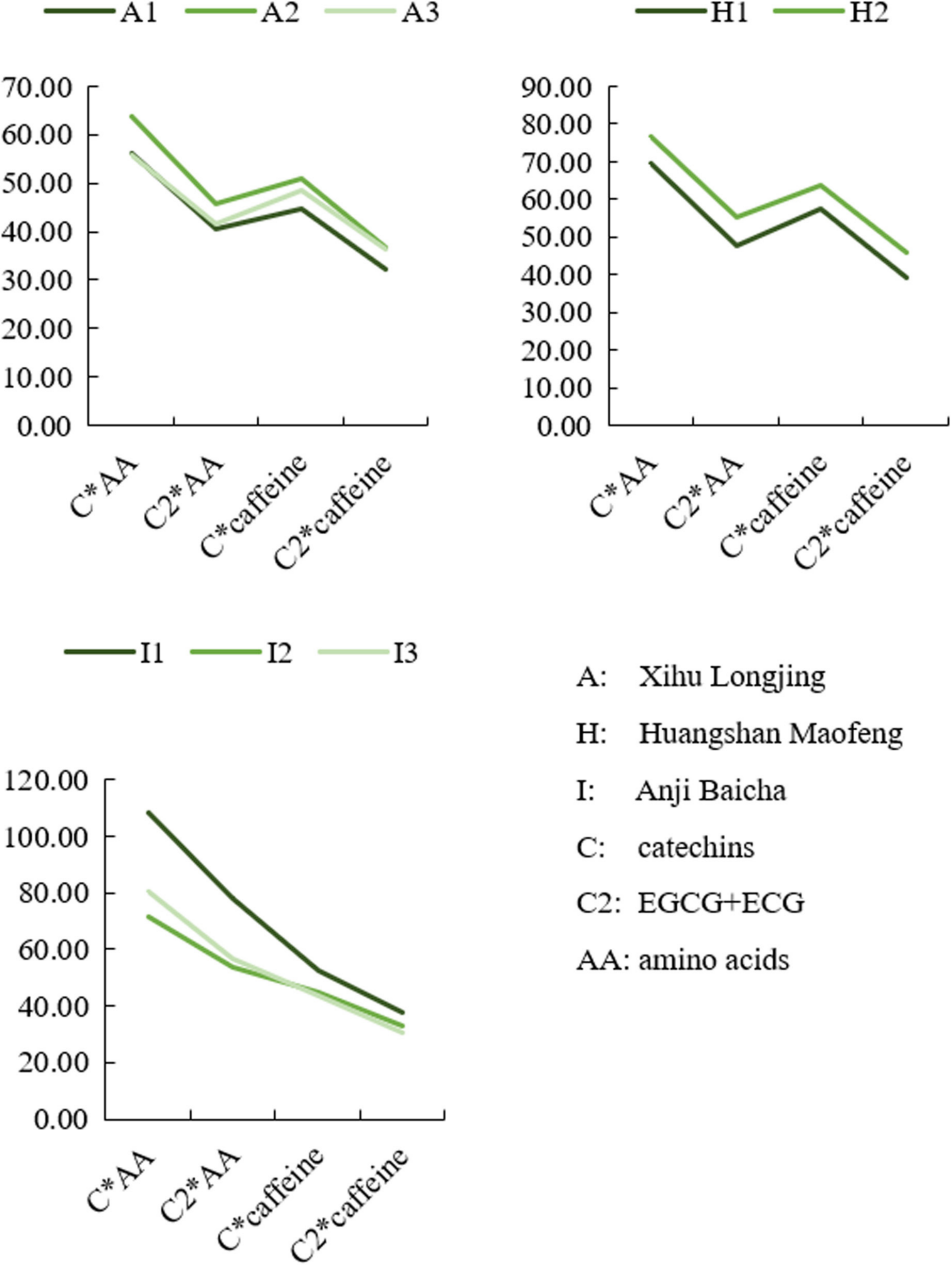
Chemical components selection for Xihu Longjing, Huangshan Maofeng, and Anji Baicha

It is clear from Figure 7 that using the content of a single chemical component to determine green tea grades is not feasible. Because for a single chemical component, such as catechins or EGCG, there is no apparent consistent relationship in its content between different grades of the same kind of green tea. However, Figure 8 shows the product of two chemical components' contents could better determine the grades in the same kind of green teas through data processing and analysis.

Figure 8 demonstrates that the ester catechins (EGCG + ECG, labeled C2 in Figure 8) play a more important role than the nonester catechins (EGC + EC) in determining green tea grades. Previous studies have confirmed that caffeine and ester catechins were the principal constituents of green tea cream (Xu & Yin, 2010). Amino acids also participated in the formation of tea cream (Lin et al., 2017; Xu & Yin, 2010), thus, the product of catechin contents and amino acid contents, as well as the product of the ester catechin contents and amino acid contents could be used to distinguish between different grades of the same kind of green tea.

The similarity between the chemical components that form green tea cream and affect green tea grades might be the underlying reason for the correlation between the turbidity difference of green tea infusions and green tea grades. Moreover, the product of two chemical components' contents might reflect that the correlation is nonlinear. In this study, C (catechins) * AA (amino acids), C2 (EGCG + ECG) * AA, C * caffeine, and C2 * caffeine were found as the effective combination for determining green tea grades (* means the multiplication sign).

## 4 CONCLUSION

In this study, a hypothesis that the turbidity difference, which reflects the amount of tea cream formed in a certain period of time, indicates the grade of green tea was proposed and tested. The hypothesis was confirmed at the preliminary stage by applying the turbidity difference as the indicator under the acceleration of Ca^2+^. Among four different types of green teas with different grades, it was found that higher grades of green tea had larger turbidity differences between the turbidities before and after the Ca^2+^ acceleration. Through the single‐variable experiments, effects of green tea brewing temperature, green tea brewing time, Ca^2+^ concentration, and Ca^2+^ treatment time were analyzed, and their optimal values were selected as the room temperature at 25°C, 20 min, 500 mg/L, and 10 min, respectively. These findings provided a potential but feasible method for simple, accessible, and on‐site determination of green tea grades; however, the sample size of green teas should have to be expanded.

## CONFLICT OF INTEREST

The authors declare that they do not have any conflict of interest.

## ETHICAL APPROVAL

This study does not involve any human or animal testing.

## INFORMED CONSENT

Written informed consent was obtained from all study participants.

## Data Availability

The data that support the findings of this study are available from the corresponding author upon reasonable request.
